# Marijuana use and sex with multiple partners among lesbian, gay and bisexual youth: results from a national sample

**DOI:** 10.1186/s12889-016-3905-0

**Published:** 2017-01-05

**Authors:** Xiaoyun Zhang, Li-Tzy Wu

**Affiliations:** 1 General Motors, Detroit, MI 48265 USA; 2 Department of Psychiatry and Behavioral Sciences, School of Medicine, Duke University Medical Center, BOX 3903, Durham, NC 27710 USA; 3 Department of Medicines, School of Medicine, Duke University, Durham, NC USA; 4 Duke Clinical Research Institute, Duke University Medical Center, Durham, NC USA

**Keywords:** Marijuana use, Sex with multiple partners, Lesbian, Gay, Bisexual youth

## Abstract

**Background:**

Sex with multiple partners (SMP) is one of the important contributing factors for contracting sexually transmitted infections (STIs) among adolescents and young adults, especially among Lesbian, Gay, and Bisexual (LGB) youth. Past studies mainly focus on examining associations of alcohol or club drugs use with unprotected sexual behaviors among adult homo/bisexual men, while little is known about the temporal association between marijuana use (MU) and SMP among LGB youth.

**Methods:**

This study examined the relationship between MU and SMP among LGB adolescents and young adults. Generalized estimating equations (GEE) logistic regression analyses were utilized to analyze four waves’ public-use Add Health data (*N* = 694, youth who reported a homo/bisexual status at any wave; Wave 1: aged 11–21; Wave 4: aged 24–32).

**Results:**

After adjusting for other substance use, current depression, mother-child relationship quality at Wave 1, and socioeconomic variables, past-year MU was both concurrently and prospectively associated with past-year SMP. The moderating effect of age was not found.

**Conclusion:**

MU is concurrently and prospectively associated with increased odds of SMP in the adolescent sample and in the young adult sample. Findings imply that prevention/intervention on HIV risk behaviors may benefit from MU reduction not only in LGB adolescents but also in young adults.

## Background

Lesbian, Gay, and Bisexual (LGB) youth are at risk for some health conditions. For example, 80% of youth aged 13–24 with a new HIV diagnosis in 2014 were males who self-identified gay or bisexual [1]. Also, lesbian and bisexual girls are more likely to report unintended pregnancy compared with their heterosexual peers [2]. Higher risks of STIs, including HIV, among homo/bisexual males and unintended pregnancy rate among lesbian and bisexual females may be related to unprotected or risky sexual behaviors such as sex with multiple partners (SMP). Compared to heterosexual youth (11.1%), a higher proportion of LGB 9th-12th graders reported having 4+ lifetime sexual partners (gay/lesbian: 29.9%; bisexual: 28.2%) [3]. To decrease risk of SMP among LGB youth, identifying risk factors to inform prevention is critical.

MU and SMP may co-occur among LGB youth. Similar to SMP, LGB youth are at risk for MU. National survey data showed that 34.5% gay/lesbian and 36.8% of bisexual 9th-12th graders reported current MU compared with 21.8% heterosexual students [1]. Further, compared to heterosexual youth, LGB youth not only had higher initial rates of substance use including use of marijuana, but the substance use prevalence also increased more rapidly during transition from adolescence to young adulthood [4]. Also, a number of studies have documented positive associations between MU and SMP among the general samples without differentiating sexual orientations [5]; further, the strength of the associations has been found to be stronger among adolescents than young adults [6]. Past studies on LGB population have focused mainly on examining associations of alcohol or club drugs use with unprotected sexual behaviors among adult homo/bisexual men [7]. However, the temporal association between MU and SMP among LGB youth is unclear. To date, many states in the United States have legalized marijuana for medical use, with some for recreational use. Legalization of MU may increase marijuana availability, which raises concerns about MU and its related problems [8]. Given high prevalence of MU [3], it is critical to understanding the relationship between MU and SMP in this young population. This study examines concurrent and prospective associations between past-year MU and SMP among LGB youth. We also examine whether the concurrent association is moderated by age group. We examine two hypotheses: 1) MU is positively associated with SMP in LGB youth; 2) the strength of the associations is stronger in adolescence than young adulthood.

## Methods

### Data source

Data were from four waves’ public-use Add Health datasets (*N* = 3342). Add Health was a longitudinal study of a national sample of 7th-12th graders during 1994–1995 academic years [9]. From September 1994 to April 1995, over 90,000 students from 132 schools (80 high schools and 52 middle schools) completed in-school questionnaires; and then the eligible students participated in-home interview (Wave 1, *N* = 20,745, age = 11–21, 79% of response rate). In 1996, students in grades 7–11 at Wave 1 were followed up for the second in-home interview (Wave 2; *N* = 14,738, age = 11–21, 88.6% of response rate). Two other in-home interviews were followed between August 2001 and April 2002 (Wave 3; *N* = 15,170, age = 18–26, 77.4% response rate), and during 2008–2009 (Wave 4; *N* = 15,701, age = 24–32, 80.3% response rate). Through in-home interviews, longitudinal survey data on the social, economic, psychological, and health circumstances were obtained, with Wave 1–2 constituting the adolescence period and Wave 3–4 focusing on the emerging and young adulthood [10]. Audio-CASI technology (audio-computer assisted self-interview) was used for sensitive health status and behavioral health-related questions to enhance the quality of self-reporting of sensitive and illegal information [10]. Study designs and procedures were described in detail in [10].

A total of 3342 participants that represented a random sample from the total sample were made available for public use (see guidelines for analyzing Add Health data, [9, 11]). The analysis sample included those who reported a homo/bisexual status at any wave (*N* = 694; 21.5% of public-use sample; 72.2% females; 68.5% non-Hispanic Whites, 12.8% non-Hispanic Blacks, 11.7% Hispanics and 6.7% non-Hispanic others). At Wave 1, 45.7% of respondents’ resident mothers and 35.6% of the fathers had 12+ years’ education; 22.3% of the families received past-month welfare. The use of de-identified Add Health datasets was declared exempt from review by the Duke University Health System Institutional Review Board.

### Measures

MU referred to “ever use” at Wave 1; and “past-year use” at Wave 2–4.

Past-year number of sexual partners referred to the number of having sexual relationships at Wave 1 and Wave 2 (since the date of last interview). At Wave 3, it referred to having vaginal intercourse; and at Wave 4, it included the number of female/male partners having sexual activities. The variables were dichotomized into having any sexual partner (ASP: 0 = 0 partner, 1 = 1+ partners) and SMP (0 = 0–1 partner, 1 = 2+ partners).

Covariates at eave wave included other types of substance use (i.e., current smoking: referring to those smoking at least 1 day during the past 30 days and ever smoking regularly; past-year alcohol use: no use, monthly use, and weekly use; other illegal drug use: lifetime use at Wave 1 and 4, past-year use at Wave 2, and past-6 years use at Wave 3, including included cocaine, inhalants, crystal meth, LSD, PCP, ecstasy, mushrooms, ice, heroin, or prescription medicines not prescribed to respondents), and current CES-D depression (9-item version of CES-D; score of 10+ indicating having depression [12]). Covariates at Wave 1 included mother-child relationship quality (5 items; 5-likert scale, the Cronbach’alpha = 0.84; scores of 20 or higher as high quality [13]), gender, age cohort, race/ethnicity, mother and father’s education level, and whether family receiving welfare. Previous findings indicate that use of other substances [7] was associated with MSP among young men who have sex with men, and that race/ethnicity [14], socioeconomic status [15], depression [16], and mother-child relationship [17] are associated with SMP among study samples without differentiating sexual orientations. We include these variables as control variables.

### Data Analysis

GEE analyses were conducted using Stata 13.0. First, we obtained concurrent unadjusted and adjusted odds ratios (AORs) and 95% confidence intervals (CIs). Second, we tested the interaction effect between MU and wave on SMP. Third, we examined whether prior MU (one-wave lagged MU) predicted increased odds of later SMP. The independent working correlation structure was specified [18]. Grand sample weights were included to make the estimates from the national sample generalizable.

## Results

Table 1 summarizes prevalence of MU and ASP/SMP. Approximately 30% of LGB youth used marijuana in the past year in 1995 and 1996. The proportions increased to 45.3% in 2001–2002, and decreased to 32.4% in 2008–2009. About 15% of LGB youth reported having more than 1 partners in 1995 and 1996. The proportions increased to more than 30% at Wave 3 (31.6%) and 4 (38.0%). Additional analyses indicated that, compared with heterosexual youth, proportions of MU in LGB youth were higher at each wave; proportions of SMP were higher at Wave 2 and 4, but not at Wave 1 and 3 (footnotes under Table 1).

**Table 1:** Weighted prevalence and 95% Confidence Interval (CI) of substance use and number of sexual partners, and weighted means and standard errors (SE) for current CES-D depression, as well as mother-child relationship at Wave 1

*N* = 694^ab^	Wave 1	Wave 2	Wave 3	Wave 4
Year of Survey	1995	1996	2001–2002	2008–2009
Age range	11–21	13–21	18–27	24–32
Prevalence % (95% CI)	% (95% CI)	% (95% CI)	% (95%CI)	% (95%CI)
^c^Past-Year Marijuana Use				
Yes	30.3(26.0–34.9)	33.9(29.8–38.3)	45.3(40.0–50.6)	32.4(28.4–36.7)
Past-Year Alcohol Use				
No use	50.0(44.9–55.2)	46.6(42.4–50.8)	23.3(18.9–28.4)	19.8(16.4–23.8)
≤ Monthly use	42.2(37.2–47.4)	41.5(37.8–45.4)	47.6(43.2–52.2)	44.7(39.9–49.6)
Weekly use	7.8(6.0–10.2)	11.8(9.5–14.6)	28.9(24.0–34.2)	35.4(30.1–41.1)
Current Smoking				
Yes	20.9(17.4–24.9)	23.4(19.8–27.4)	39.9(35.2–44.9)	43.5(39.1–48.0)
Ever Other Illegal Drug Use				
Yes	18.2(14.1–23.1)	13.3(10.5–16.7)	42.0(37.3–46.8)	50.0(45.2–55.0)
Past-Year Having Any Sexual Partners				
Yes	24.0(19.9–28.7)	21.2(17.7–25.3)	77.9(73.4–81.8)	88.9(86.1–91.2)
^d^Past-Year Having Multiple Sexual Partners				
Yes	15.9(13.0–19.4)	15.7(12.7–19.4)	31.6(27.8–35.8)	38.0(32.8–43.4)
Current CES-D Depression				
Mean (SE)	6.62(6.23–7.00)	6.89(6.52–7.25)	5.97(5.60–6.34)	6.61(6.21–7.02)
Cronbach’ alpha	0.80	0.83	0.84	0.83
Mother-Child Relationship-W1				
Range	8–25			
Mean (SE)	20.6(20.3–21.0)			
Cronbach’alpha	0.85			

^a^Homo/bisexual respondents, *n* = 694: at Wave 1 and 2, Add Health data provided at most nine sexual partners’ gender information for each respondent. Another question was added to ask about if respondents had any other partners additionally. Respondents who had at least one same-sex partner were identified as homo/bisexual cases. At Wave 3, those who identified themselves totally homosexual, mostly homosexual, equally homosexual or heterosexual, mostly heterosexual, were defined as homo/bisexual cases. At Wave 4, similar to Wave 3, those who did not identified themselves as totally heterosexual were defined as homo/bisexual cases. Furthermore, respondents who reported the number of same-sex partners (even 0) were identified as homo/bisexual cases

^b^The percentages of missing data on the studied variables ranged from 0 to 1.4%, except for mother-child relationship quality (4.8%, mostly due to no resident mother at home), and number of sexual partners at Wave 4 (5.8%, program error)

^c,d^We used “svy: tab” commands in Stata to examine whether the proportions of marijuana use (MU) and sex with multiple partners (SMP) at each wave were significantly different between homo/bisexual youth and their heterosexual counterparts. The design-based results were following: MU: *F*(1130) = 12.17 (Wave 1); *F*(1130) = 24.55 (Wave 2); *F*(1130) = 21.07 (Wave3); *F*(1130) = 37.15 (Wave 4); *p* < .001. SMP: *F*(1130) = 2.32, *p* > .05 (Wave 1); *F*(1130) = 6.75, *p* < .05 (Wave 2); *F*(1130) = 1.74 (Wave 3); *p* > .05; *F*(1130) = 24.63, *p* < .001. Prevalence (%) of MU from Wave 1 to 4 among heterosexual youth: 22.9, 23.4, 32.9, 21.4; SMP: 13.6; 11.8; 28.6; 23.7

In the unadjusted analysis, past-year MU was associated with past-year SMP (OR = 2.79, 95% CI = 2.22, 3.51; Table 2). Past-year MU remained modestly associated with increased odds of SMP after adjusting for covariates (AOR = 1.55, 95% CI = 1.18, 2.04; Table 3). The interaction effect of MU and wave on SMP was insignificant (Table 4). Further analysis indicated that prior MU was modestly associated with increased odds of later SMP (AOR = 1.42, 95%CI = 1.07, 1.89; Table 5).

**Table 2:** Generalized estimating equations (GEE): Unadjusted longitudinal logistic regression analysis of each studied variable and number of sexual partners (OR and 95% CI, *N* = 694)

Independent variables	Having Any Sexual Partner (Wave 1 to 4)	Having Multiple Sexual Partners (Wave 1 to 4)
Past-Year Marijuana Use		
	2.15(1.79–2.59)^c^	2.79(2.22–3.51)^c^
Past-Year Alcohol Use		
≤ monthly use vs. no use	2.77(2.22–3.47)^c^	2.21(1.68–2.89)^c^
weekly use vs. no use	6.80(5.12–9.04)^c^	5.05(3.72–6.86)^c^
Current Smoking		
	3.48(2.84–4.26)^c^	2.97(2.35–3.74)^c^
Ever Other Illegal Drug Use		
	4.33(3.51–5.33)^c^	3.05(2.48–3.75)^c^
Current CES-D (≥10 = 1)	1.33(1.08–1.64)^b^	1.49(1.17–1.90)^b^
Mother-Child Relationship-W1(≥20 = 1)	0.71(0.60–0.85)^c^	0.75(0.58–0.96)^a^
Gender (male = 1)	0.88(0.72–1.07)	1.25(0.97–1.61)
Age group in years		
15–17 vs. 11–14	1.46(1.24–1.72)^c^	1.37(1.09–1.74)^b^
18^+^ vs. 11–14	2.06(1.45–2.94)^c^	1.38(0.83–2.28)
Race/Ethnicity		
Hispanic vs. Non-Hispanic White	1.11(0.89–1.39)	0.80(0.54–1.19)
Non-Hispanic Black vs. Non-Hispanic White	1.57(1.25–1.98)^c^	1.82(1.38–2.39)^c^
Non-Hispanic Others vs. Non-Hispanic White	1.06(0.76–1.48)	1.50(0.93–2.42)
Respondent’s Mother Education Level		
college or more vs. high school and below	0.79(0.67–0.93)^b^	0.92(0.72–1.17)
missing vs. high school and below	0.97(0.71–1.32)	1.04(0.69–1.56)
Respondent’s Father Education Level		
college or more vs. high school and below	0.85(0.69–1.04)	0.96(0.72–1.28)
missing vs. high school and below	1.04(0.84–1.29)	1.10(0.82–1.48)
Family Receiving Welfare-W1		
yes vs. no	0.98(0.79–1.21)	1.08(0.82–1.41)
missing vs. no	1.01(0.80–1.28)	0.99(0.72–1.38)

^a^< .05

^b^< .01

^c^< .001

**Table 3:** Generalized estimating equations (GEE): Adjusted longitudinal logistic regression analysis of marijuana use and number of sexual partners (AOR and 95% CI, *N* = 694^ab^)

Independent variables	Having Any Sexual Partner (Wave 1 to 4)	Having Multiple Sexual Partners (Wave 1 to 4)
Past-Year Marijuana Use		
	0.85(0.65–1.10)	1.55(1.18–2.04)^b^
Past-Year Alcohol Use		
≤ monthly use vs. no use	2.32(1.82–2.97)^c^	1.81(1.36–2.41)^c^
weekly use vs. no use	4.96(3.48–7.07)^c^	3.37(2.42–4.69)^c^
Current Smoking		
	2.33(1.77–3.06)^c^	1.87(1.42–2.47)^c^
Ever Other Illegal Drug Use		
	3.05(2.34–3.98)^c^	1.91(1.47–2.47)^c^
^c^Current CES-D(≥10 = 1)	0.84(0.64–1.11)	1.16(0.85–1.57)
^d^Mother-Child Relationship-W1(≥20 = 1)	0.89(0.70–1.12)	0.94(0.70–1.25)
Gender (male = 1)	0.82(0.65–1.04)	1.23(0.93–1.63)
Age group in years		
15–17 vs. 11–14	1.29(1.05–1.57)^a^	1.17(0.90–1.51)
18^+^ vs. 11–14	1.62(1.02–2.55)^a^	0.81(0.45–1.48)
Race/Ethnicity		
Hispanic vs. Non-Hispanic White	1.17(0.91–1.49)	0.81(0.56–1.17)
Non-Hispanic Black vs. Non-Hispanic White	2.76(2.08–3.66)^c^	3.09(2.18–4.40)^c^
Non-Hispanic Others vs. Non-Hispanic White	1.08(0.73–1.61)	1.66(0.95–2.88)
Respondent’s Mother Education Level		
college or more vs. high school and below	0.84(0.67–1.04)	0.89(0.67–1.19)
missing vs. high school and below	0.77(0.45–1.32)	0.80(0.48–1.32)
Respondent’s Father Education Level		
college or more vs. high school and below	0.75(0.58–0.98)^a^	0.83(0.60–1.15)
missing vs. high school and below	0.90(0.69–1.16)	0.87(0.62–1.20)
Family Receiving Welfare-W1		
yes vs. no	0.83(0.66–1.04)	0.99(0.72–1.35)
missing vs. no	0.72(0.53–0.98)^a^	0.97(0.67–1.39)

^a^< .05

^b^< .01

^c^< .001

**Table 4:** Generalized estimating equations (GEE): Testing the interaction effect between marijuana use and wave on number of sexual partners (AOR and 95% CI, *N* = 694)

Independent variables	Having Any Sexual Partner (Wave 1 to 4)	Having Multiple Sexual Partners (Wave 1 to 4)
Past-Year Marijuana Use	1.34(0.76–2.36)	2.34(1.43–3.81)^b^
Wave (Wave1 = 0)	3.84(3.20–4.62)^c^	1.50(1.27–1.76)^c^
Past-Year Marijuana Use × Wave		
	1.01(0.74–1.37)	0.87(0.69–1.09)
Past-Year Alcohol Use		
≤ monthly use vs. no use	1.70(1.23–2.33)^b^	1.56(1.17–2.09)^b^
weekly use vs. no use	2.10(1.39–3.18)^c^	2.51(1.81–3.49)^c^
Current Smoking		
	1.93(1.37–2.71)^c^	1.68(1.27–2.22)^c^
Ever Other Illegal Drug Use		
	1.82(1.34–2.49)^c^	1.54(1.16–2.05)^b^
Current CES-D(≥10 = 1)	1.04(0.76–1.44)	1.24(0.92–1.66)
Mother-Child Relationship-W1(≥20 = 1)	0.78(0.59–1.04)	0.92(0.69–1.24)
Gender (male = 1)	0.79(0.58–1.08)	1.24(0.93–1.64)
Age group in years		
15–17 vs. 11–14	1.62(1.24–2.10)^c^	1.20(0.92–1.55)
18^+^ vs. 11–14	2.26(1.15–4.45)^a^	0.87(0.46–1.63)
Race/Ethnicity		
Hispanic vs. Non-Hispanic White	1.13(0.81–1.58)	0.80(0.55–1.18)
Non-Hispanic Black vs. Non-Hispanic White	2.83(1.92–4.16)^c^	2.89(2.02–4.12)^c^
Non-Hispanic Others vs. Non-Hispanic White	1.02(0.60–1.75)	1.66(0.95–2.92)
Respondent’s Mother Education Level		
college or more vs. high school and below	0.71(0.53–0.95)^a^	0.87(0.65–1.16)
missing vs. high school and below	0.59(0.31–1.10)	0.77(0.46–1.31)
Respondent’s Father Education Level		
college or more vs. high school and below	0.78(0.55–1.11)	0.86(0.62–1.20)
missing vs. high school and below	0.94(0.67–1.31)	0.89(0.64–1.24)
Family Receiving Welfare-W1		
yes vs. no	0.73(0.53–1.01)	0.99(0.72–1.35)
missing vs. no	0.66(0.45–0.99)^a^	0.99(0.68–1.43)

^a^< .05

^b^< .01

^c^< .001

**Table 5:** Generalized estimating equations (GEE): Adjusted longitudinal logistic regression analysis of one-wave lagged effect of marijuana use and number of sexual partners (AOR and 95% CI, *N* = 694)

Independent variables	Having Any Sexual Partner (Wave 1 to 4)	Having Multiple Sexual Partners (Wave 1 to 4)
Lagged (Past-Year Marijuana Use)		
	1.35(1.01–1.81)*	1.42(1.07–1.89)*
Past-Year Alcohol Use		
≤ monthly use vs. no use	2.04(1.57–2.67)***	1.91(1.38–2.63)***
weekly use vs. no use	3.42(2.33–5.02)***	3.41(2.39–4.87)***
Current Smoking		
	2.12(1.56–2.88)***	1.70(1.28–2.27)***
Ever Other Illegal Drug Use		
	2.92(2.18–3.90)***	1.81(1.36–2.40)***
Current CES-D(≥10 = 1)	0.76(0.55–1.05)	1.25(0.91–1.71)
Mother-Child Relationship-W1(≥20 = 1)	0.90(0.71–1.15)	0.96(0.70–1.31)
Gender (male = 1)	0.80(0.61–1.04)	1.36(1.00–1.85)
Age group in years		
15–17 vs. 11–14	1.20(0.97–1.48)	0.98(0.74–1.29)
18^+^ vs. 11–14	1.21(0.68–2.16)	0.71(0.37–1.40)
Race/Ethnicity		
Hispanic vs. Non-Hispanic White	1.30(0.98–1.73)	0.83(0.55–1.25)
Non-Hispanic Black vs. Non-Hispanic White	2.78(2.03–3.80)***	2.99(2.07–4.31)***
Non-Hispanic Others vs. Non-Hispanic White	1.16(0.75–1.79)	1.95(1.11–3.44)*
Respondent’s Mother Education Level		
college or more vs. high school and below	0.84(0.67–1.07)	0.91(0.67–1.24)
missing vs. high school and below	0.94(0.50–1.76)	0.82(0.47–1.41)
Respondent’s Father Education Level		
college or more vs. high school and below	0.85(0.63–1.14)	0.93(0.65–1.31)
missing vs. high school and below	1.03(0.78–1.37)	1.00(0.70–1.42)
Family Receiving Welfare-W1		
yes vs. no	0.83(0.65–1.07)	1.06(0.77–1.47)
missing vs. no	0.76(0.52–1.10)	1.04(0.73–1.48)

* < .05, *** < .001

## Discussion

This study examined the temporal relationship between MU and SMP in a national sample of LGB youth. Past-year MU was concurrently and prospectively associated with increased odds of past-year SMP after adjusting for covariates, and the strength of concurrent associations was similar in the adolescent sample and the young adult sample. It implies that prevention/intervention on SMP may benefit from MU reduction in both LGB adolescents and young adults. These findings are inconsistent with those found from the general population sample that included both heterosexual and homo/bisexual youth in the analysis, as the later found that the strength of associations between MU and SMP decrease by age as adolescence transitioned to young adulthood [6]. This analysis provided new findings for LGB youth because prior studies have focused on adult homo/bisexual men, in which significant associations between MU and unprotected sexual behaviors were inconsistently found [7]. Specifically, prior studies tended to employ convenience samples drawn from men attending gay bars/clubs or sex parties, who might use various illicit drugs with sex [7]. Our sample was drawn from a national sample of LGB youth, thus findings may have a greater generalizability than results from convenience samples. Additionally, we examined the association of MU and SMP in a younger LGB sample. Focusing on adolescents and young adults is important to inform prevention. Early MU and SMP may have a devastating impact on health. For example, prevalence MU disorder among users of MU is generally higher among adolescents/youth than adults [19]. Early SMP is linked with STIs, unwanted pregnancies, and poverty [20]. Early prevention/intervention is critically needed to prevent adverse outcomes in adulthood.

Using the data from a national sample, we found that the prevalence of past-year MU and SMP among LGB youth were higher than those among heterosexual youth, suggesting that LGB youth are at higher risk for MU and SMP [3]. Two theories may explain the link between MU and SMP [21]. First, MU may increase risk for SMP through impairing young users’ decision-making or judgement. Second, MU and SMP may be influenced by certain shared risk factors (e.g., LGB-relate stressors, other substance use). Compared with heterosexual youth, LGB youth reported a higher prevalence of mental health problems [22], childhood sexual/physical abuse, and peer victimization [23]. They may engage in self-sabotaging behaviors like MU and SMP to escape from these traumatic issues and emotional distress [24]. Future studies may test the two theories simultaneously using one comprehensive model, or test possible mechanisms (e.g., MU outcome expectancy) between MU and SMP.

The study has limitations. SMP was not measured consistently across waves. It may help to explain no significant differences found in the proportions of SMP between heterosexual and LGB youth at Wave 1 and 3. Interpretation of results should be with caution. Researchers should measure the same construct consistently across measurement points. Second, this study relied on self-report measures of MU and SMP, potential measurement errors and age-related changes in reporting behaviors might exist and confound the results [6]. Thirdly, we only used past-year SMP as an indicator of risky sexual behaviors. If youth who have multiple partners at a given time but use condoms consistently, they are at a lower risk for contracting STIs. However, available data indicate that having a higher number of sexual partners is associated with a lower prevalence of condom use [25]. Thus, SMP may serve as one of the important indicators of risky sexual behaviors. Despite these limitations, this study is among the first to examine the temporal relationship between MU and SMP over time among LGB youth.

## Conclusions

In conclusion, this study examined both the concurrent association and prospective association between past-year MU and past-year SMP in a national LGB adolescents and young adults. Findings indicate that past-year MU is both concurrently and prospectively associated with past-year SMP, while the concurrent associations between the two do not vary over time. Prevention and intervention efforts aimed at reducing SMP in LGB youth may benefit from reduction of MU both in adolescence and young adulthood.

## Abbreviations

ASP: Having any sexual partner
CES-D: Center for Epidemiologic Studies Depression Scale
GEE: Generalized estimating equations
LGB: Lesbian, gay and bisexual
MU: Marijuana use
SMP: Sex with multiple partners
STIs: Sexually transmitted infections
